# On the potential of agentic workflows for animal training plan generation

**DOI:** 10.3389/fvets.2025.1563233

**Published:** 2025-05-20

**Authors:** Jörg Schultz

**Affiliations:** Tier Wohl Team GbR, Rödelsee, Germany

**Keywords:** computer assisted animal training, LangGraph, agent orchestration, modularity in AI systems, welfareaware AI systems, training plan customization, handler support tools, task-specific training workflows

## Abstract

Effective animal training depends on well-structured training plans that ensure consistent progress and measurable outcomes. However, the creation of such plans is often time-intensive, repetitive, and detracts from hands-on training. Recent advancements in generative AI powered by large language models (LLMs) provide potential solutions but frequently fail to produce actionable, individualized plans tailored to specific contexts. This limitation is particularly significant given the diverse tasks performed by dogs–ranging from working roles in military and police operations to competitive sports–and the varying training philosophies among practitioners. To address these challenges, a modular agentic workflow framework is proposed, leveraging LLMs while mitigating their shortcomings. By decomposing the training plan generation process into specialized building blocks–autonomous agents that handle subtasks such as structuring progressions, ensuring welfare compliance, and adhering to team-specific standard operating procedures (SOPs)—this approach facilitates the creation of specific, actionable plans. The modular design further allows workflows to be tailored to the unique requirements of individual tasks and philosophies. As a proof of concept, a complete training plan generation workflow is presented, integrating these agents into a cohesive system. This framework prioritizes flexibility and adaptability, empowering trainers to create customized solutions while leveraging generative AI's capabilities. In summary, agentic workflows bridge the gap between cutting-edge technology and the practical, diverse needs of the animal training community. As such, they could form a crucial foundation for advancing computer-assisted animal training methodologies.

## 1 Introduction

As a professional animal trainer, creating structured training plans is not merely a helpful tool but a foundational practice that enhances the effectiveness and consistency of training. These plans serve two essential purposes: they provide a clear roadmap for progressive skill development and act as vital documentation for tracking progress and ensuring accountability. However, despite their undeniable value, the process of writing training plans can be both time-consuming and repetitive. Trainers often find themselves devoting significant effort to tasks that involve structuring similar steps for different contexts, leaving less time for the hands-on interaction and observation essential to effective training. Streamlining the creation of these plans could enable trainers to dedicate more time to working directly with their animals, improving outcomes while reducing the burden of administrative work.

Recent advancements in artificial intelligence, particularly in the field of generative AI, offer a solution by supporting processes such as writing training plans. Generative AI refers to a category of machine learning models capable of producing coherent and creative outputs, including text, code, and even images, by leveraging patterns learned from vast datasets (1–3). These models, powered by large language models (LLMs) like OpenAI's GPT, have demonstrated remarkable capabilities across a diverse range of tasks. For instance, generative AI simplifies complex programming tasks by generating code snippets or even entire programs ^1^. Additionally it has also excelled in intellectually demanding areas, such as high-stakes competitions like the International Biology Olympiad (4), where it has provided accurate and contextually appropriate answers. These achievements highlight the adaptability and problem-solving capacity of generative AI, making it a compelling candidate for assisting in the creation of training plans (5).

Still, relying on standard chatbots like ChatGPT to produce training plans currently reveals significant limitations. Although the generated plans may read well, they often lack the specificity required for actionable progressions with clearly defined steps. Furthermore, such plans are typically generic and may fail to adhere to agency-specific training standards, including critical welfare considerations. Standard prompting approaches do not incorporate standard operating procedures (SOPs), leaving a gap in consistency and compliance with established guidelines. Additionally, these chatbots cannot effectively interact with trainers to iteratively refine goals or ensure that all relevant information–such as the animal's health conditions or unique circumstances–is taken into account. These shortcomings highlight the need for a more robust, structured approach to harness the capabilities of generative AI for creating truly actionable and individualized training plans.

To inform the development of such an approach, it is instructive to examine how human experts construct training plans in practice. A closer analysis of established methodologies in both companion and working dog training can provide valuable insights into the modularity, sequencing, and contextualization that characterize effective plan design.

Although developing an effective training plan is a nuanced and context-dependent process, several common principles can be identified across domains. In professional companion dog training, the process typically begins with a comprehensive assessment phase^2^.

Trainers collect relevant background information through structured intake forms and in-depth interviews to understand the dog's behavior history, living conditions, and the owner's training goals. This step may be followed by additional research on the target behavior, especially when dealing with complex or atypical issues. Based on the gathered information, trainers formulate a training roadmap that outlines key behavioral objectives. These objectives are then decomposed into smaller, teachable components, often structured into modular lesson plans^3^. Each session includes specific success criteria, which may relate to duration, distance, environmental distractions, or generalization across contexts. Trainers frequently document these plans with written instructions for homework and progress tracking to ensure consistency between training sessions and reinforce client adherence. For example, protocols from the *Manual of Clinical Behavioral Medicine for Dogs and Cats* (6) specify steps as detailed as “Sit for 15 s” or “Sit while you take two steps backward and return,” illustrating the level of precision and progression often required in practice^4^. Such structured and measurable criteria are critical for ensuring clarity and success–especially when training is conducted by clients outside of formal sessions. This level of specificity, however, is rarely achieved through standard chatbot-generated plans, which tend to produce generalized advice rather than clearly staged, actionable instructions.

In the context of working dogs–such as those in guide dog programs, search-and-rescue operations, or military and police K9 units–training is even more structured. These programs typically follow well-defined curricula that progress through sequential training phases^5^.

Trainers begin with a clearly defined end-goal behavior (e.g., reliable indication of explosives or successful navigation in a guide dog context) and systematically break it down into a series of intermediate goals. Each of these may have its own mini-plan, addressing specialized components such as odor imprinting, alert behavior, or environmental generalization. The training plans are closely tied to institutional Standard Operating Procedures (SOPs), which ensure consistent application across handlers and maintain high standards of animal welfare and operational reliability.

This structured, modular, and often collaborative methodology reveals a key insight: the development of training plans by human experts is inherently a multi-step process. Rather than generating complete plans in a single action, trainers systematically address a sequence of interdependent subtasks, each focused on a specific component of the training context. This observation holds important implications for AI-assisted training plan generation. If the objective is to replicate the quality, clarity, and contextual relevance of plans produced by experienced professionals, then a single, monolithic language model query is insufficient. Instead, a more viable approach involves decomposing the overall task into discrete stages and assigning each to a specialized system component–mirroring the sequential, modular workflow employed by human practitioners.

Each of these system components can be implemented using a large language model (LLM), tailored to address a narrowly defined subtask within the broader training plan development process. Because each subtask is limited in scope–such as defining progression steps, selecting appropriate criteria, or integrating welfare guidelines–the quality and specificity of the generated output can be significantly improved compared to a single, monolithic query. In the terminology of artificial intelligence, such a specialized unit is referred to as an *agent*: an autonomous module designed to perform a specific function. To ensure that the outputs of these agents align coherently and contribute to a unified, actionable training plan, their interactions must be carefully coordinated. This process of managing the communication and sequencing between multiple agents is referred to as an *agentic workflow* (7). Just as a human trainer must ensure that the individual components of a training plan build on one another in a logical and effective manner, agentic workflows aim to replicate this coordination through structured, modular interactions among AI agents.

The concept of agentic workflows is not just theoretical; it is a rapidly emerging field with promising applications across diverse domains. For example, the *ChatDev* system demonstrates the power of communicative agents in software development (8). This workflow orchestrates specialized agents to collaboratively perform tasks such as designing, coding, and testing software, mimicking a streamlined development team. Similarly, in the realm of human behavior simulation, *Generative Agents: Interactive Simulacra of Human Behavior* showcases how agentic workflows can create lifelike simulations, enabling detailed studies of complex interactions between autonomous agents in virtual environments (9). Another compelling example is the *Agent Hospital*, which employs medical agents to simulate hospital operations, allowing for the exploration of adaptive strategies in healthcare systems (10). In each case, the modularity, adaptability, and task specialization of agentic workflows have proven instrumental in addressing domain-specific challenges efficiently and effectively. These examples illustrate the significant potential of agentic workflows to tackle complex, multifaceted challenges by decomposing them into manageable tasks. If agentic workflows have proven so effective in domains such as software development, human behavior simulation, and healthcare operations, it is compelling to explore whether they could also address the challenge of generating training plans. Given the modularity and adaptability of these workflows, their application to training plan creation could open new possibilities for improving both efficiency and quality in animal training.

This manuscript aims to explore the potential of agentic workflows for training plan generation by presenting a team of AI agents capable of creating individualized, actionable training plans. However, the goal is not to deliver a rigid, monolithic, one-size-fits-all solution. The rapid pace of technological advancements, particularly in generative AI, means static workflows risk becoming quickly outdated. Additionally, the range of tasks performed by dogs is vast, training philosophies vary, and different training teams have unique SOPs. To address these challenges, this work introduces adaptable building blocks–autonomous agents and teams of agents–that tackle discrete aspects of training plan generation. The modularity of these building blocks enables seamless integration of new AI models or refined task descriptions, allowing the system to evolve with future advancements without requiring a complete overhaul. Furthermore, these building blocks are intentionally designed for flexibility, enabling users to modify, combine, and repurpose them to meet the specific requirements of their training units or organizations. By empowering trainers and organizations to customize workflows, this approach ensures it remains relevant and responsive to evolving standards, technologies, and needs.

## 2 Method

### 2.1 Agentic frameworks

The implementation of agentic workflows is greatly facilitated by specialized frameworks designed to coordinate multi-agent systems. These frameworks streamline the development, deployment, and orchestration of agents, enabling effective collaboration on complex tasks. Table 1 summarizes several widely used frameworks, highlighting their key features and suitability for different applications.

**Table 1:** Comparison of selected AI-agent frameworks.

**Framework**	**Summary**	**Special features**
AutoGen^*a*^	A Microsoft framework for building collaborative multi-agent systems with shared memory and task execution.	Offers strong integration with memory systems and facilitates natural agent collaboration.
CrewAI^*b*^	A framework specializing in team-based agent workflows, where agents take on defined roles and responsibilities.	Focuses on role-based collaboration, making it suitable for task-specific teamwork and simulations.
LangGraph^*c*^	A graph-based agent orchestration framework designed for controlled, iterative workflows and safe AI interactions.	Modular design allows flexible and programmable workflows, ideal for research and safety-critical domains.
OpenAI Swarm^*d*^	A framework enabling the deployment of multiple cooperative AI agents using OpenAI's API. Agents interact to solve complex tasks collaboratively.	Emphasizes scalability and efficient task distribution in multi-agent setups.

^a^ https://github.com/microsoft/autogen

^b^ https://github.com/crewAIInc/crewAI

^c^ https://github.com/langchainai/langchain

^d^https://github.com/openai/swarm.

For this work, *LangGraph* was chosen due to its emphasis on control, reproducibility, and adaptability–qualities essential for generating training plans for living organisms, such as dogs. LangGraph's graph-based architecture enables precise orchestration of agent interactions, allowing each step in the workflow to be explicitly defined, monitored, and adjusted as needed. This transparency ensures that the process remains consistent, traceable, and aligned with ethical standards. Unlike other frameworks that prioritize rapid iteration or emergent behaviors, LangGraph provides fine-grained control over agent operations, minimizing the risk of unintended actions. Its modular design supports the incorporation of domain-specific constraints, such as welfare principles and progression guidelines, ensuring that outputs meet the stringent requirements of animal training. Furthermore, LangGraph facilitates automated testing at each stage of the workflow, validating intermediate outputs and ensuring compliance with predefined standards, which enhances both safety and reproducibility.

These features make LangGraph particularly suited for applications involving living beings, where safety, predictability, and ethical considerations are paramount. Additionally, its adaptability allows for seamless integration of new technologies or evolving training methodologies, ensuring that the system can grow and improve without requiring a complete redesign.

### 2.2 Agent implementation

#### 2.2.1 Core agent

**Figure 1:**
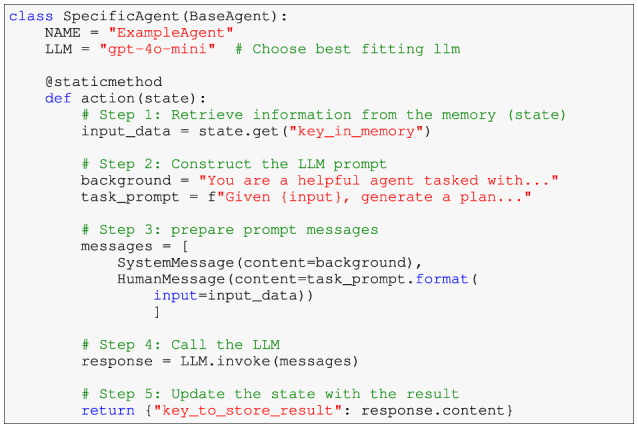
Template for a typical agent implementation — The example demonstrates the workflow for retrieving input from memory, constructing an LLM prompt, invoking the LLM, and updating the agent's state with the result.

The agentic workflow is built upon a modular architecture, where each agent is designed to handle a specific subtask of the training plan generation process. To ensure consistency and adherence to defined coding standards, all agents inherit from a shared base class (Figure 1). This base class enforces a uniform structure and provides essential functionality, such as naming and action definition.

The *BaseAgent* class ensures that each agent:

- Defines a unique NAME attribute to identify its role within the workflow.
- Implements a static action method to perform its primary task.
- Includes optional utility methods to enable interaction in different environments.

Each agent follows a standardized workflow that consists of three main steps:

1. State interaction: retrieve relevant information from the shared memory or state.
2. Prompt construction: prepare input for the large language model (LLM). This often includes a background story (system message) and a specific task prompt (human message).
3. LLM invocation and output handling: call the LLM to generate output and update the shared memory with the result.

This design ensures modularity, allowing agents to be independently developed, tested, and integrated into the broader workflow. By leveraging the modular capabilities of LangGraph, these agents can collaborate seamlessly, with their interactions explicitly defined and traceable.

#### 2.2.2 Enhancing agent functionality

Agents in this workflow are designed to handle specific tasks autonomously. Their functionality is significantly enhanced through two key features: the integration of external tools and the use of structured output formats. Together, these enhancements improve the adaptability, efficiency, and reliability of the system.

One critical aspect of agent functionality is their ability to autonomously determine when external tools are necessary to complete their tasks. **Tools** provide agents with access to additional resources or data, enabling them to refine outputs, address knowledge gaps, and adapt to dynamic requirements (11) . For example, data retrieval tools allow agents to query external resources, such as databases or online repositories, to gather up-to-date and task-specific information. Similarly, interaction-based tools enable agents to request clarification or additional input from human collaborators or other agents, facilitating problem-solving in scenarios where initial input may be incomplete or ambiguous. By analyzing the input data, agents can dynamically decide which tools to invoke, ensuring efficient operation while avoiding unnecessary or redundant usage.

Another essential feature of the workflow is the use of **structured output** formats. Unlike free-form text, which can vary across iterations or models, structured outputs adhere to predefined formats such as JSON or XML. This approach offers several benefits: structured outputs ensure consistency across multiple runs, even when generative models introduce randomness; they enable machine-readable results that can be directly parsed and utilized by other agents or systems; and they simplify validation processes, ensuring that outputs comply with task-specific requirements. These attributes make structured outputs vital for creating robust, modular workflows where multiple agents collaborate seamlessly.

By combining autonomous tool usage with structured output mechanisms, the system achieves a high degree of reliability and scalability. These features enable agents to perform their tasks effectively while ensuring that their outputs integrate seamlessly into the broader workflow. Together, these enhancements demonstrate the potential of agentic systems to address complex, real-world challenges with precision and adaptability.

#### 2.2.3 Agent collaboration

The true power of the agentic workflow emerges from interactions between agents, elevating the system beyond the capabilities of individual agents (12). By collaborating in well-defined ways, agents can tackle complex tasks with efficiency and adaptability. Several interaction patterns can be employed, each suited to specific scenarios:

- **Sequential collaboration**: a straightforward interaction where each agent's output serves as the input for the next in a predefined sequence. This approach ensures a logical progression from initial input to final result, making it ideal for tasks requiring step-by-step refinement.
- **Conditional edges**: agents can dynamically invoke other agents or teams based on the results of their tasks. This enables adaptive workflows that respond to varying input data or scenarios, ensuring flexibility in achieving desired outcomes.
- **Reviewer pattern**: in this iterative approach, one agent reviews the output of another, providing feedback or suggestions for improvement (13). The original agent refines its output based on the review and resubmits it for evaluation. This pattern is particularly useful for tasks demanding high precision and specialization.
- **Sub graphs as teams**: groups of agents form specialized sub-teams (sub-graphs), each focusing on a specific aspect of the overall task. These teams work independently, sharing selected information with the larger workflow. This modular structure simplifies complex tasks while maintaining controlled information flow.
- **Router (manager)**: a central management agent directs tasks to the most appropriate team or agent based on predefined rules or contextual factors. This pattern is well-suited for workflows involving diverse subtasks or requiring task prioritization.
- **Map reduce**: for tasks that can be divided into smaller, independent subtasks, this pattern enables parallel execution. A coordinator agent distributes the subtasks among agents, collects their outputs, and combines them into the final result. This approach is particularly effective for managing large or complex tasks efficiently (14).
- **Shared memory**: agents share information by accessing and modifying a centralized memory (15). Separate memories can be used for different teams, with selected portions shared across the workflow. This maintains modularity while ensuring coherence in information exchange.

These interaction patterns, ranging from straightforward sequential workflows to dynamic and adaptive processes, enable agentic systems to efficiently address complex challenges. Selecting the appropriate pattern depends on task complexity, the need for adaptability, and the desired level of modularity and control.

### 2.3 Testing and validation

Rigorous testing is a cornerstone of reliable software development, ensuring that individual components and systems function as intended (16, 17). However, when building agent frameworks that integrate large language models (LLMs), testing becomes even more critical. LLMs introduce a probabilistic element to the workflow, where outputs can vary based on subtle changes in input or model state. This variability makes testing essential, particularly when developing modular building blocks (agents) that need to function consistently across diverse scenarios.

In this workflow, testing must validate not only individual agents but also their interactions within teams. To achieve this, a multi-level testing strategy was employed:

1. **Unit testing:** unit tests validate the functionality of individual agents in isolation. These tests ensure that each agent interacts correctly with its dependencies, such as invoking a mocked LLM with the expected parameters. They also verify the agent's internal logic and state management, ensuring that intermediate outputs conform to the expected structure and logic. For example, a unit test might confirm that the correct input is passed to a mocked LLM and that the output adheres to predefined specifications.
2. **Integration testing:** integration tests assess the ability of agents to interact with real external systems, particularly LLMs. These tests verify that the LLM processes input data and produces responses with the expected structure or features. For example, an integration test might validate that the output includes key elements relevant to the task and adheres to the required format. Integration tests provide confidence in the agent's ability to function effectively in real-world scenarios.
3. **Probabilistic output validation:** given the inherent variability in LLM outputs, exact matches with expected results are not always feasible. To address this, a validation step leverages another LLM call to evaluate outputs against predefined criteria. Instead of requiring verbatim equality, the validating LLM assesses whether the response includes essential elements and adheres to the desired format. This approach ensures robust testing while accounting for the probabilistic nature of LLM-generated outputs.

This three-tiered testing strategy ensures that agents and their interactions perform reliably. Unit tests provide a foundation for correctness by validating the internal logic and mocked interactions of individual agents. Integration tests confirm that agents operate effectively in real-world scenarios, interacting seamlessly with LLMs and producing structured outputs. Finally, probabilistic validation accommodates the inherent variability of LLMs by focusing on alignment with predefined criteria, ensuring robustness and flexibility. By adopting this comprehensive testing approach, the development of high-quality, reusable building blocks is supported. This strategy ensures that agents and teams function predictably, even as input conditions or details change, thereby reinforcing the modular and adaptable nature of the proposed workflow.

### 2.4 Code availability

The software developed for this study, AI Agents for Animal Training Plan Generation, is available at GitHub^6^. The software is platform-independent and has been tested on Windows, and Linux operating systems. It is primarily written in Python and requires Python version 3.11 or higher due to dependencies associated with LangGraph. Users should ensure they have the appropriate Python version installed before running the software. The project requires several Python packages, which are listed in the requirements.txt file within the repository.

The repository includes a comprehensive suite of tests to validate both individual agents and their interactions. These tests can be executed using the “pytest” framework with specific markers for test selection: “pytest-m unit” runs unit tests, “pytest-m integration” runs integration tests, and “pytest-m llm” executes probabilistic output validation tests. The tests vary in runtime and cost depending on their complexity and external resource usage. Unit tests are fully local and execute quickly, as they mock dependencies and avoid external API calls. Integration tests, by contrast, involve calls to the LLM whenever required by the agent, leading to moderate runtimes and associated costs depending on the number of tests. Probabilistic output validation tests, which include an additional LLM call to analyze the results of the agent's LLM interaction, have the longest runtimes and highest costs. This flexibility allows users to select specific testing levels based on their requirements and resource constraints.

The software is licensed under the MIT License, which permits use, distribution, and modification for both academic and non-academic purposes. Detailed instructions for installation, setup, and usage are available in the README.md file within the repository^7^. Users must obtain an OpenAI API Key to utilize the default language model gtp-4o-mini and a Tavily API Key if employing the Internet Research Agent.

The version of the software used in this manuscript is tagged as v1.0.0 in the repository.

## 3 Results

### 3.1 Agents: core components of the workflow

#### 3.1.1 A minimalistic agent design-tackling distraction training

As outlined in the introduction, training plans are typically organized into modular lesson units, each targeting specific success criteria such as duration, distance, or resistance to distractions. Distraction-proofing is a particularly important objective, especially when dogs are expected to perform reliably in unpredictable or high-stimulus environments. Within the agentic workflow, this training goal is addressed by a dedicated *Distraction Specialist Agent*, which generates lesson plans focused on gradually increasing distraction levels while maintaining behavioral performance.

**Figure 2:**
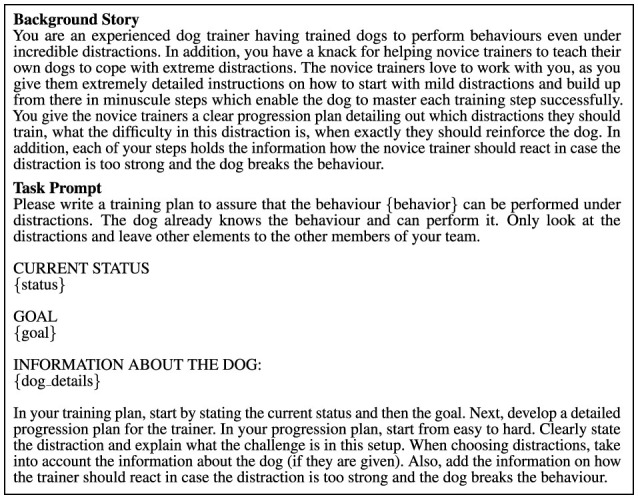
Example of a prompt for an agent — This specific prompt is designed for the *Distraction Specialist Agent* and guides it in generating a progression plan to ensure reliable performance of a trained behavior under distractions. It includes the behavior, current status, goal, and relevant dog details, ensuring the generated plan is detailed, step-by-step, and tailored to the dog's needs and the trainer's actions.

From a technical perspective, the *Distraction Specialist Agent* (full code in Supplementary material, Section 1.1.1) exemplifies the foundational principles of the agentic workflow. It demonstrates how structured inputs, targeted prompts, and the use of a large language model (LLM) can produce actionable and specialized outputs. This agent is designed to address a common and critical challenge in dog training: ensuring that a trained behavior can be reliably performed even under distractions. Its workflow is both straightforward and effective, following a sequence of key steps. First, the agent accesses relevant information from the shared memory, including the behavior being trained, the current status of the dog, the desired goal, and any specific details about the dog's circumstances. Using this input, the agent crafts a prompt that combines a detailed background story with a task-specific instruction, clearly defining the LLM's role in generating a tailored training plan (Figure 2).

The task prompt is designed to guide the LLM in generating highly structured and actionable outputs. By explicitly defining sections such as “CURRENT STATUS”, “GOAL”, and “INFORMATION ABOUT THE DOG” the prompt ensures that the response aligns with the expectations of the training plan framework. Additionally, the instruction to progress from easy to hard distractions provides a logical structure to the training plan, ensuring clarity and usability for novice trainers. Specific guidance, such as reacting to distractions that are too strong, further personalizes the output and prepares trainers for real-world scenarios. An example of a training plan generated by the agent, with the initial status of “Dog can sit when the trainer moves their arms” and the goal of “Dog stays sitting when a ball is thrown,” is provided in the Supplementary material, Section 1.1.2.

The response generated by the LLM is stored in the shared memory as a draft_plan, making it accessible to other agents as needed. This interaction with the memory not only enables seamless communication between agents but also ensures that the output remains adaptable for review or refinement by other specialized agents, such as the *Welfare Agent*. This design guarantees consistency and adaptability, enabling the *Distraction Specialist Agent* to handle a variety of distraction-related scenarios while aligning seamlessly with the overall agentic workflow strategy.

However, the *Distraction Specialist Agent's* simplicity also presents a limitation: it relies entirely on the LLM's output without leveraging any additional sources or predefined guidelines. This dependence on a single source may reduce consistency or adherence to specific standards when generating plans. In the next section, I explore how integrating internal information, such as SOPs, can enhance an agent's ability to produce outputs that align with established best practices.

#### 3.1.2 Incorporating external information and SOPs — Adding duration to a behavior

In many working dog programs, training plans are not only modular but also governed by institutional Standard Operating Procedures (SOPs), which define precise progression steps for building behaviors such as duration, distance, or environmental generalization. These SOPs ensure consistency across trainers and uphold established welfare and performance standards. Automating training plan generation in such contexts therefore requires more than generic prompting; it must incorporate domain-specific guidelines that reflect structured, validated protocols. Within the agentic workflow, nadling this need is exemplified by the *Duration Specialist Agent*, which generates progression-based lesson plans while adhering to predefined SOPs.

At the implementation level, the *Duration Specialist Agent* extends the foundational agentic design by incorporating external domain knowledge into the training plan generation process. Unlike the *Distraction Specialist Agent*, which relies solely on prompt engineering, this agent accesses standardized progression steps stored in configuration files. These steps, derived from established training guidelines such as those described in Spector's work on obedience shaping (18), are selected based on the current status and goal defined in shared memory, then integrated into the LLM prompt to guide the output. A key advantage of this design is its modularity: by simply editing the configuration file, users can adapt the agent's behavior without altering the underlying code. This allows trainers or domain experts–regardless of programming experience–to modify progression strategies in alignment with their institutional standards. This adaptability further highlights the potential of the workflow to integrate domain-specific knowledge while remaining accessible to a broader audience.

To ensure that the LLM output aligns with predefined guidelines, the agent uses single-shot prompting (19). That is, by including an example output format in the prompt, the agent informs the LLM about the expected structure of the response. This approach maintains flexibility while reducing variability in the generated outputs, helping to ensure that the progression steps are accurately reflected in the final training plan. As a result, a plan generated by this agent can look like this:

1. **33.0 s:**

- Start with 33 s.
- Repeat with 16 s.
- 5 s
- 24 s
- 45 s
- 1 s
- 16 s
- 8 s
**...**
**2. 45.0 s:**
- Start with 45 s.
- Repeat with 22 s.
**...**

This design demonstrates how internal information, such as SOPs, can be incorporated into an agent's workflow to improve consistency and adherence to standards. However, this approach remains relatively simple, relying on a static text based configuration file for predefined progressions. Future enhancements could involve more dynamic methods, such as Retrieval-Augmented Generation (RAG) (20) , where the agent retrieves SOPs or other relevant guidelines from a large knowledge base in real time. Such advancements would allow agents to handle a wider range of tasks and adapt to evolving contexts with even greater precision.

### 3.2 Collaborative agent interactions: building modular teams

#### 3.2.1 Conditional edges — Collecting information dynamically

Before writing a training plan, a human trainer must develop a clear understanding of the behavior to be trained. In many cases, this understanding is based on prior experience; in others, it may require additional research or clarification. Trainers may consult external sources–such as literature, protocols, or online resources–or seek further input from the handler to better understand the context, motivation, or constraints associated with the behavior. Within the agentic workflow, this process is mirrored by the *Outline Writer Agent*, which is responsible for drafting an initial plan outline and determining whether the available information is sufficient to proceed.

**Figure 3:**
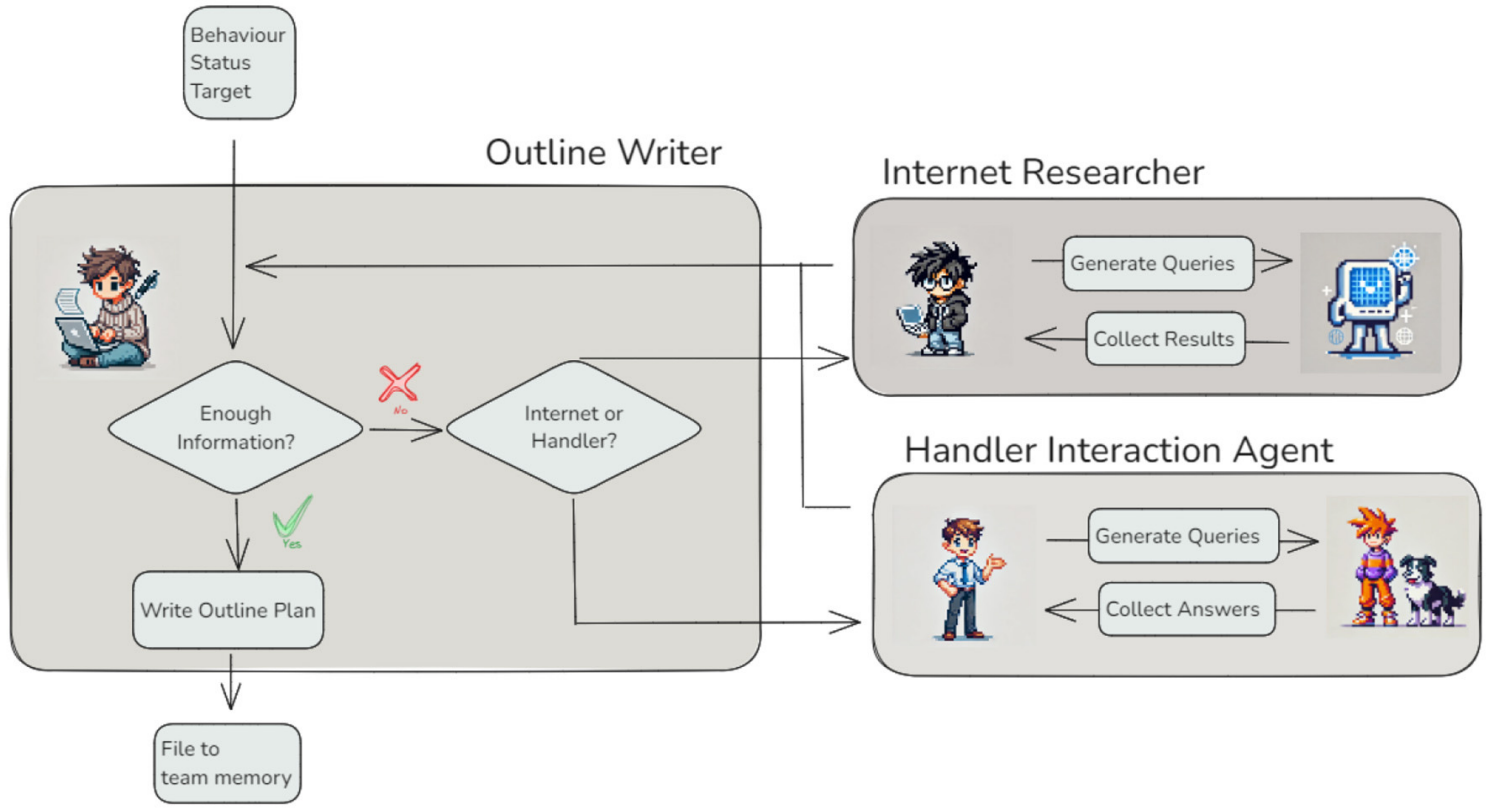
Conditional edges in agent collaboration — When insufficient information is available, the *Outline Writer Agent* dynamically determines whether to consult the *Internet Researcher Agent* or the *Handler Interaction Agent*, based on the nature of the missing data. Each supporting agent performs its specialized task, returning results to the *Outline Writer Agent* for generating a complete outline plan.

To do so, the agent must be capable of making decisions dynamically, based on the current state of input and task requirements. This capability is achieved through the concept of conditional edges, which enable agents to decide the next step in their workflow rather than following a predefined sequence of actions. Conditional edges introduce flexibility and autonomy, elevating agents from simple task executors to intelligent decision-makers. The *Outline Writer Agent* (Figure 3) provides an excellent example of this functionality. Its primary task is to generate a plan outline based on the given behavior, current status, and target goal. However, before proceeding, the agent evaluates whether it has sufficient information to complete the task. If additional input is required, the *Outline Writer Agent* determines whether to request it from the *Internet Researcher Agent* or the *Handler Interaction Agent*. This decision depends on the nature of the missing information–whether it can be sourced from online references or requires input directly from the dog handler.

This example highlights the core idea of agency: the ability of agents to make decisions autonomously. By incorporating conditional edges, agents not only perform predefined tasks but also adapt to dynamic conditions, making the workflow more robust and intelligent.

#### 3.2.2 Map reduce and structured output — Breaking down training steps

While conditional edges enable agents to make decisions dynamically, they are limited when workflows require managing multiple tasks simultaneously or activating an unknown number of additional agents. This limitation becomes particularly relevant once a high-level training roadmap has been established. In practice, a human head trainer would review the roadmap, identify the specific training components needed, and delegate each to a specialist or team for detailed implementation. Similarly, in the agentic workflow, the task of breaking down a plan outline into actionable components and coordinating their distribution is handled by the *Team Manager Agent*. Since the number and nature of these steps are not always known in advance, it becomes necessary to identify and manage them dynamically. This challenge is effectively managed using a Map-Reduce approach–a strategy that divides tasks into smaller, manageable units, processes them in parallel, and efficiently recombines the results (14).

**Figure 4:**
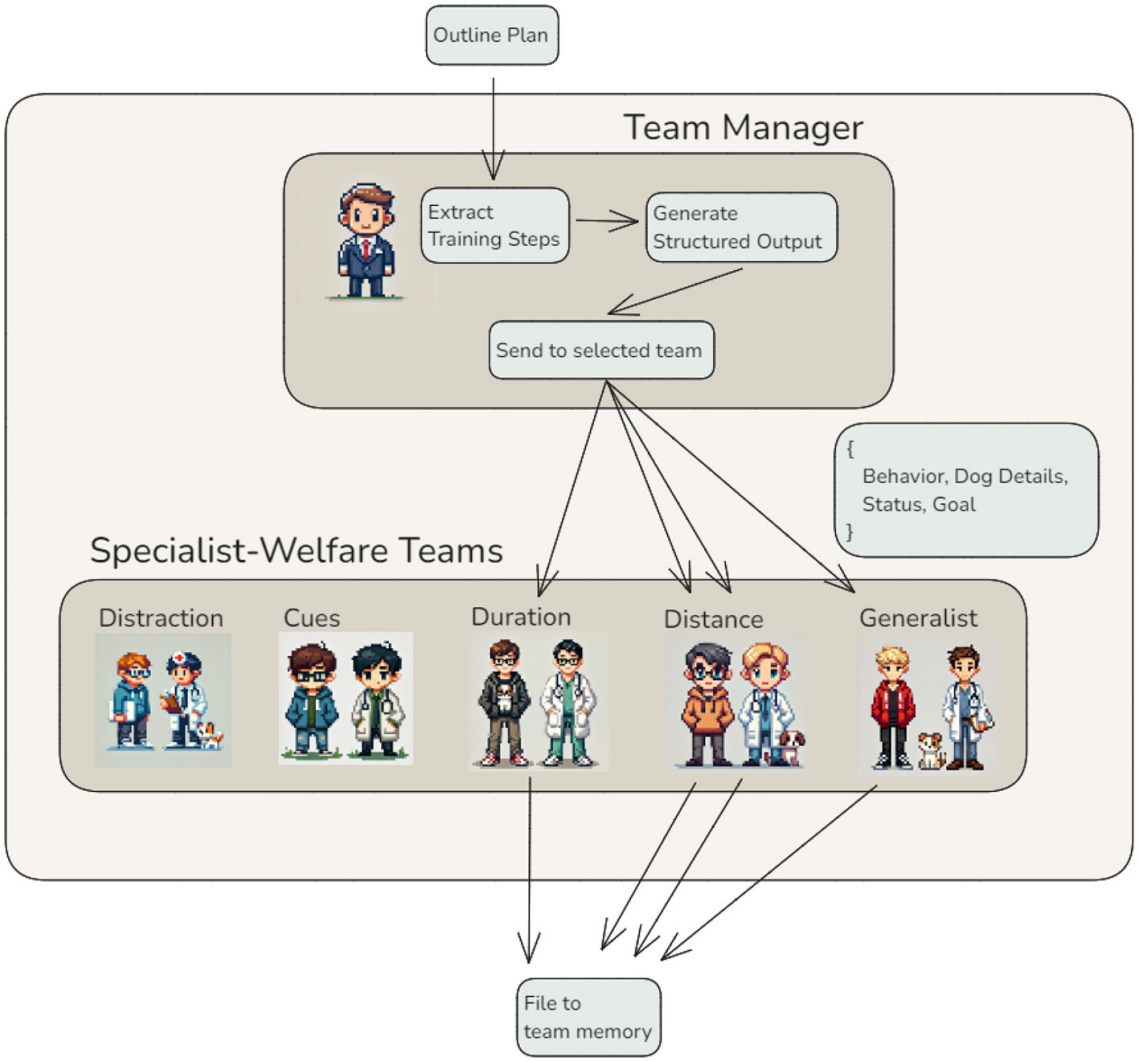
Map-Reduce workflow with structured output — The *Team Manager Agent* orchestrates a Map-Reduce pattern for training plan generation. Individual training steps are extracted from the outline plan, structured as JSON outputs, and distributed to the Specialist-Welfare Teams based on the specific requirements of each step. Each team processes its task independently and stores the results in shared memory, ensuring modularity, scalability, and consistency in the workflow.

The identification and distribution of tasks are implemented in the *Team Manager Agent* (Figure 4). Starting with a training plan outline, this agent extracts individual training steps and determines which team of agents is best suited to handle each step. To achieve this, the agent leverages the capabilities of a large language model (LLM) in a novel way: instead of generating plain text outputs, it prompts the LLM to produce structured outputs in JSON format. This structured format explicitly defines each training step, the corresponding team, and additional parameters required to complete the task. Structured outputs are particularly advantageous in complex workflows, as they ensure that accurate and complete information is transmitted to subsequent agents, avoiding ambiguities inherent in natural language responses. This guarantees consistency and reliability when activating multiple teams. Once the training steps and their details are extracted, the *Team Manager Agent* dynamically activates the necessary Specialist-Welfare Teams. Each team processes its assigned task independently and writes its output directly to the shared memory. Importantly, the teams work in parallel, significantly reducing the time required to generate the final training plan while preserving the modularity and scalability of the system. After all teams complete their tasks, their outputs are collected from the shared memory for further integration.

This example demonstrates a higher level of complexity in agentic workflows, showcasing how structured outputs, specialized teams, and parallel processing enhance the system's modularity, flexibility, and scalability. By leveraging Map-Reduce principles, this approach addresses the limitations of conditional edges, enabling the system to dynamically adapt to diverse and complex scenarios while ensuring high-quality outputs.

#### 3.2.3 Reviewer agents — Refining training plans for welfare compliance

**Figure 5:**
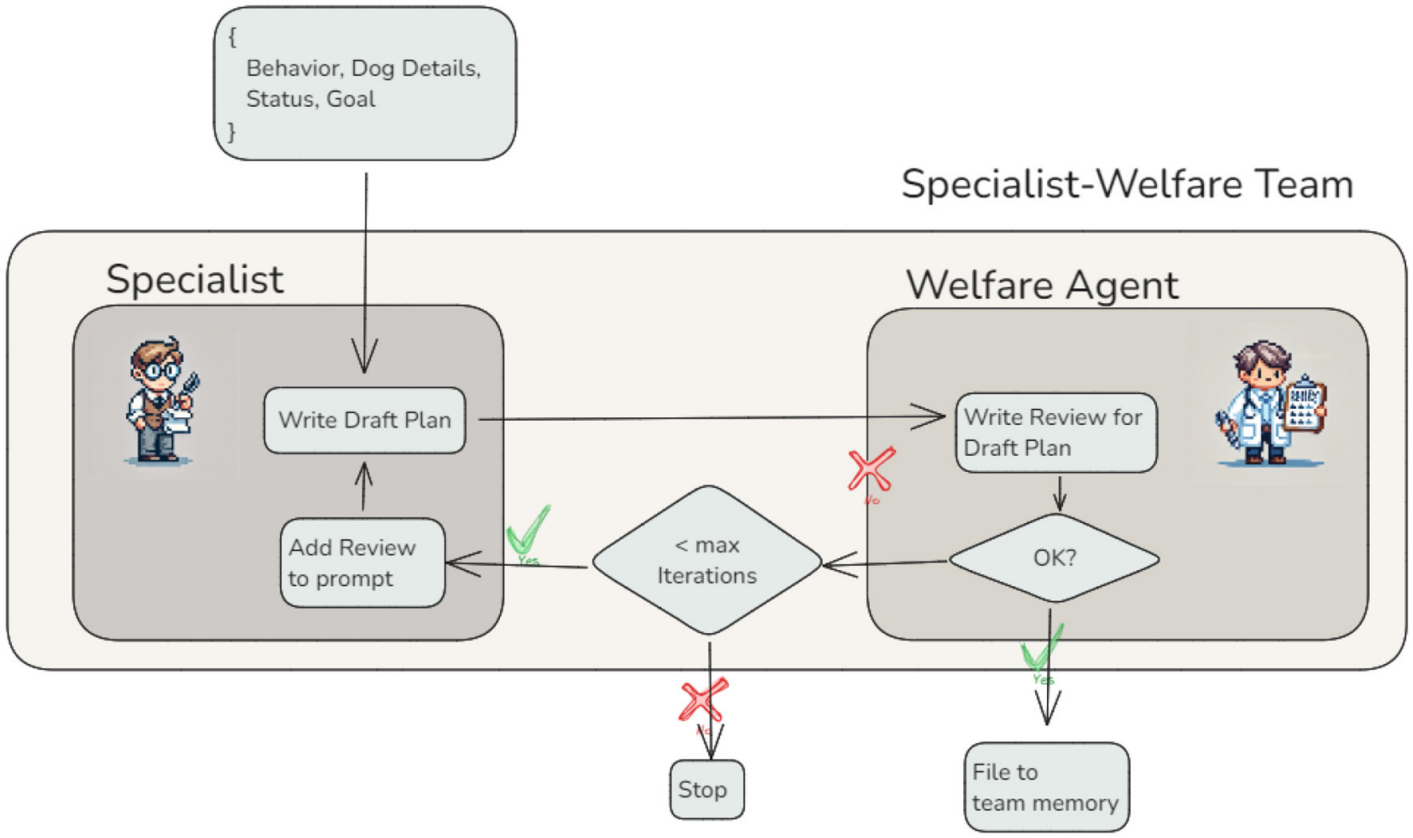
Refining LLM results with review agents — Review agents, such as the *Welfare Agent*, improve the quality of outputs through iterative evaluation and feedback. In this workflow, the Specialist Agent drafts a training plan, which the *Welfare Agent* reviews for compliance with welfare standards. Feedback is incorporated iteratively, with a maximum iteration limit to prevent infinite loops.

The integration of a Reviewer Agent introduces a new dimension to agent interactions by enabling iterative refinement of outputs (13). This is exemplified by the *Welfare Agent*, which collaborates with Specialist Plan Writers, such as the *Duration Specialist Agent*, to ensure that training plans align with predefined welfare principles (Figure 5). This architecture highlights two critical features of agent orchestration: the ability to incorporate expert feedback loops and the potential for iterative cycles within the workflow. The *Welfare Agent* does not modify the training plan directly. Instead, it evaluates the plan against encoded welfare principles and provides a detailed review, including specific suggestions for improvement. For instance, the agent may flag ethical or practical concerns and recommend adjustments to ensure the plan adheres to humane training practices. This review is then handed back to the Specialist Plan Writer, who updates the plan based on the feedback. The revised plan is resubmitted to the *Welfare Agent* for further evaluation, allowing iterative refinement. This process is illustrated through two integration tests. In the first case, the draft plan includes the following step:

**Part of draft plan**

Use a leash correction to enforce the sit position.

**Review**

Feedback: the use of a leash correction is considered an aversive method and is not in line with positive reinforcement training principles. Instead, I recommend using treats or praise to encourage the dog to sit. This approach will promote a more positive learning environment and ensure the dog's wellbeing. Please revise this step to align with humane training practices.

In the second test case, the draft plan addressed a French Bulldog with known breathing problems:

**Part of draft plan**

Get the dog motivated by throwing a ball and let him run a lot.

**Review**

Feedback: while getting the dog motivated is important, throwing a ball for a French Bulldog, especially one with potential breathing issues, may not be the best approach. Instead, consider using treats or toys that require less physical exertion to avoid putting strain on the dog's respiratory system. Ensure that the motivation method aligns with the dog's health status to prevent any discomfort or health risks.

These cases illustrate the *Welfare Agent's* ability to identify potential issues in training plans and provide constructive feedback aligned with welfare principles. In both examples, the agent flagged concerns–whether ethical, practical, or health-related–and suggested humane and effective alternatives. This feedback is then handed back to the Specialist Plan Writer, ensuring that the revised plans address the identified issues while adhering to best practices. To prevent infinite loops or excessively long cycles, the workflow includes a safeguard: a maximum iteration limit. This ensures that the process terminates after a defined number of cycles, even if the *Welfare Agent* is not fully satisfied with the plan. The final iteration results are stored in the shared memory for use by other agents or for presentation to the user.

This design demonstrates the flexibility and robustness of agentic workflows by introducing dynamic cycles as a core feature of agent interactions. By separating review and revision tasks into distinct agents, this architecture promotes modularity, scalability, and clarity–particularly in workflows that involve complex and multidisciplinary requirements.

### 3.3 Integrating tools: expanding agent capabilities

#### 3.3.1 Internet research agent — Gathering web-based background knowledge

Even experienced trainers occasionally encounter unfamiliar behaviors, edge cases, or novel training contexts that require additional research. In such situations, they may consult published literature, training protocols, or trusted online resources to supplement their knowledge before finalizing a plan. To mimic this aspect of human decision-making, the agentic workflow incorporates a mechanism for accessing external information sources: the *Internet Research Agent*.

In the previous sections, agents relied primarily on large language models (LLMs) to generate outputs. However, integrating external tools can expand their functionality and adaptability to meet more diverse requirements. The *Internet Research Agent* demonstrates this integration by leveraging the internet to gather additional, up-to-date, and context-specific information (Figure 3). Rather than simply executing a basic search query, this agent employs a systematic approach to ensure that the retrieved information is both relevant and well-structured. This ensures that the collected data can be efficiently utilized by other agents within the workflow.

The *Internet Research Agent* receives the behavior to train from the *Outline Writer Agent*. Instead of directly searching for it, the agent first leverages an LLM to refine the query according to predefined training styles and generate variations to ensure comprehensive results. Specifically, the agent adapts the query to align with positive reinforcement principles and exclude aversive methods. It also generates multiple variations of the query to explore diverse perspectives and approaches, increasing the likelihood of retrieving relevant and informative results. For each derived question, the agent performs an internet search and retrieves a predefined number of results. It then scrapes these webpages, and adds the content to the shared memory thereby making it accessible to other agents. By using this multi-step process, the *Internet Research Agent* ensures that the retrieved data is both comprehensive and tailored to the needs of the workflow. Importantly, this tool-based approach enables the system to address scenarios where the LLM alone cannot provide sufficient or up-to-date information.

This example demonstrates the versatility of agentic workflows when augmented with external tools. The *Internet Research Agent* effectively bridges the gap between real-time data access and the LLM's generative capabilities, providing other agents with a richer context to enhance their outputs.

#### 3.3.2 Reasoning and acting — Iterative goal refinement with the handler

Before developing a training plan, human trainers routinely gather detailed information from the dog's handler. This typically involves structured intake forms and follow-up conversations to clarify the dog's behavioral history, current capabilities, and specific training goals. In the agentic workflow, this step is replicated by the *Handler Interaction Agent*, which is responsible for collecting and refining handler-provided input to ensure that downstream agents can work with well-defined goals and context.

**Figure 6:**
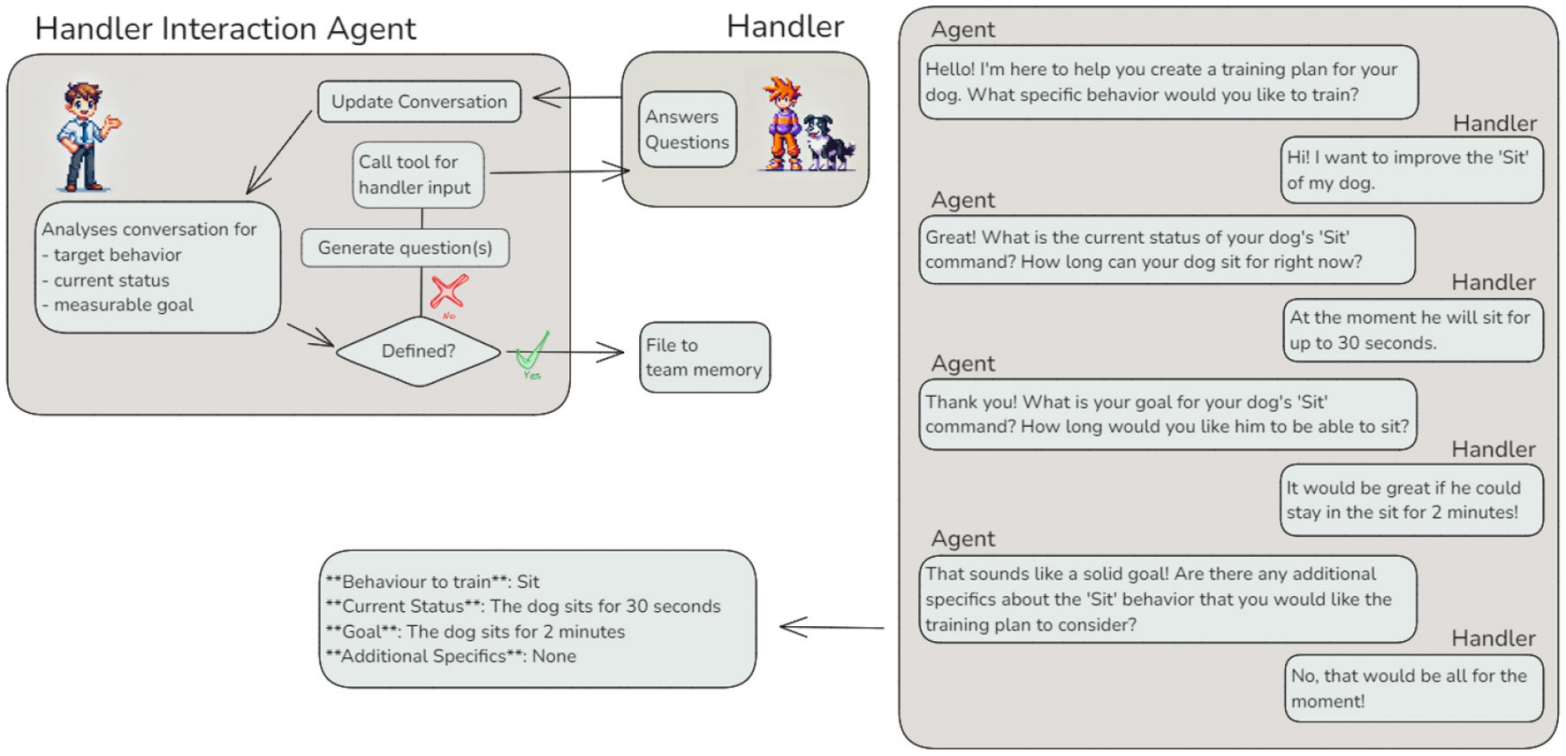
Reasoning and Acting (ReAct) with tool usage — The *Handler Interaction Agent* exemplifies the ReAct architecture by iteratively refining its understanding of a training plan goal through interaction with a human handler, treated as a “tool”. The left panel illustrates the agent's workflow, dynamically generating questions and updating the conversation based on handler input. The right panel shows an example interaction between the agent and the handler, culminating in a well-defined training plan goal.

The *Handler Interaction Agent* leverages the ReAct (Reasoning and Acting) architecture (21), representing a significant advancement in tool usage. Unlike agents with predefined workflows, this agent dynamically determines the number of interactions required with the handler to achieve its task (Figure 6).

To refine a training plan goal, the *Handler Interaction Agent* begins by analyzing the input memory to evaluate whether the behavior, current status, and goal are sufficiently defined. If the input is incomplete or unclear, the agent formulates targeted questions to request clarification or additional details from the handler. These questions bridge the gap between ambiguous input and the detailed requirements of subsequent agents. Using the handler as a “tool,” the agent iteratively refines its understanding by continuously assessing the completeness of the input and updating the conversation. This reasoning-action loop continues until the necessary details are provided or predefined interaction limits are reached. By dynamically determining the required number of interactions, the agent ensures precise goal definitions, even in scenarios where the handler may initially provide vague or incomplete objectives. A sample interaction is shown in Figure 6.

This iterative approach highlights the importance of dynamic, handler-driven refinement in agentic workflows. By ensuring that ambiguous input is clarified and detailed objectives are established, the *Handler Interaction Agent* plays a critical role in bridging the gap between vague initial input and the precise specifications required by subsequent agents. This capability not only enhances the accuracy of downstream tasks but also exemplifies the adaptability and autonomy introduced by integrating the ReAct architecture into agentic systems.

### 3.4 An integrated workflow: generating comprehensive training plans

**Figure 7:**
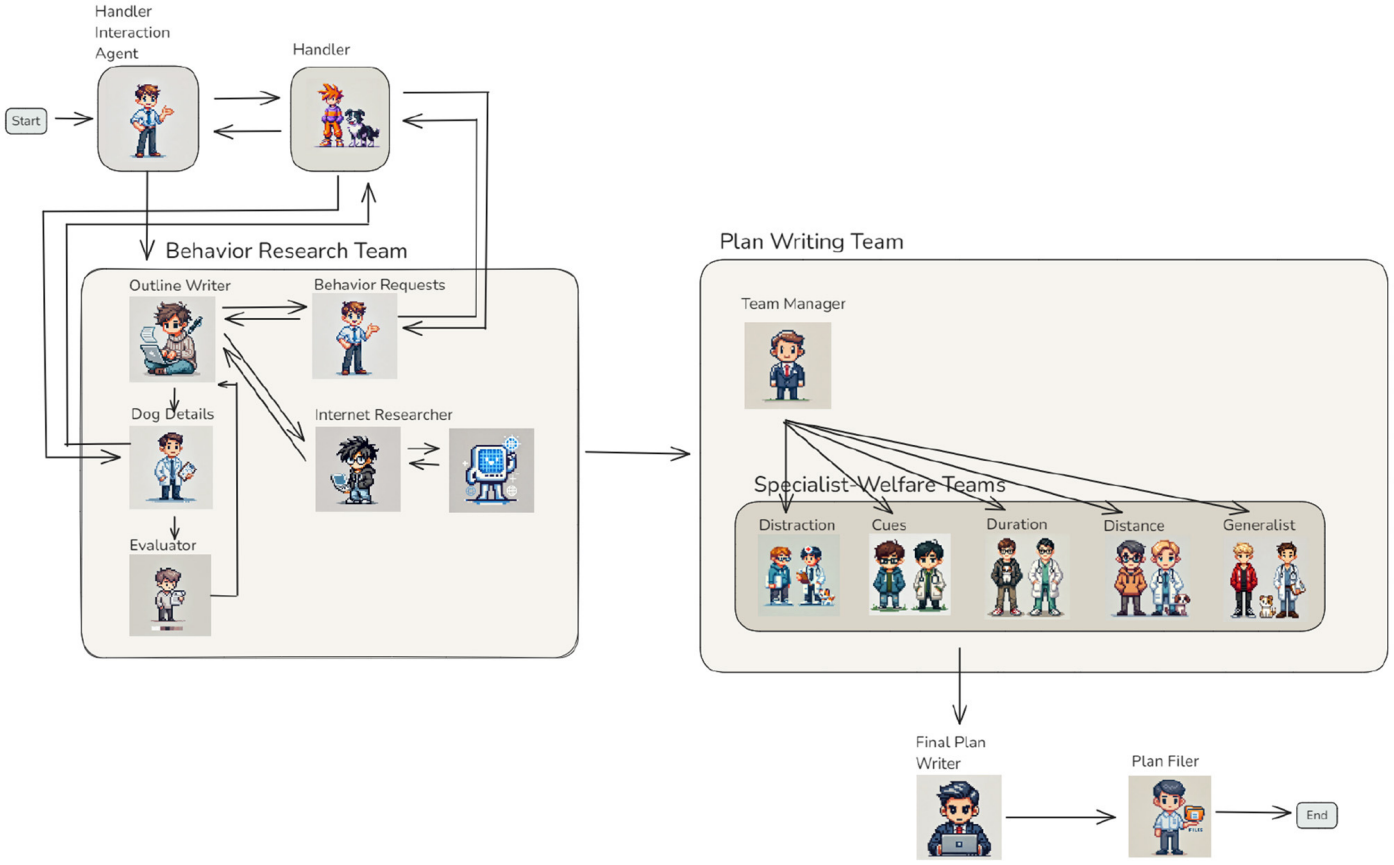
Complete workflow of the training plan team — This sample workflow integrates all agents and teams into a comprehensive system for generating detailed training plans. The process begins with the *Handler Interaction Agent*, which collects input from the handler to define the behavior, current status, and goal. This information flows into the *Behavior Research Team*, where agents such as the *Outline Writer, Internet Researcher*, and *Evaluator* collaborate to refine the outline plan. The refined outline is passed to the *Plan Writing Team*, led by the *Team Manager*, who distributes tasks to Specialist-Welfare Teams based on the specific requirements of each training step. The workflow concludes with the *Final Plan Writer* and *Plan Filer*, who consolidate and store the completed training plans, ensuring modularity, scalability, and precision in the system.

Building on the individual agents and teams introduced in the previous sections, this section presents how they can be orchestrated into a cohesive workflow capable of generating detailed training plans tailored to specific behaviors and individual dogs (Figure 7). The workflow begins with the *Handler Interaction Agent*, which gathers essential information directly from the handler. This includes defining the target behavior, its current status, and a measurable training goal. Once this information is clarified, the *Behavior Research Team* takes over.

**Figure 8:**
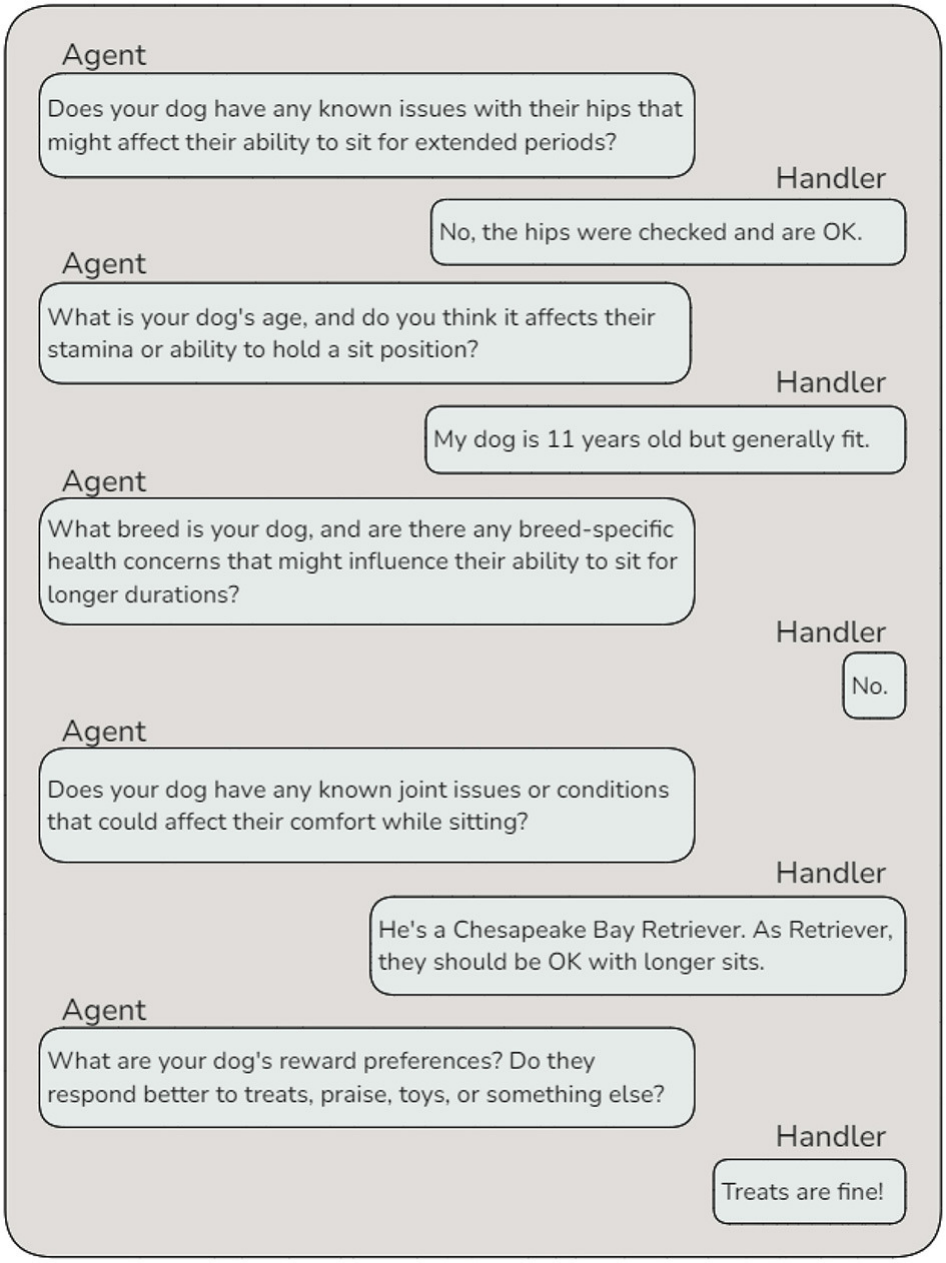
Agent-handler interaction example — This dialogue demonstrates the interaction between the *Dog Details Agent* and the handler. The agent dynamically generates specific, goal behavior relevant questions to gather information about the dog's health, breed-specific concerns, and reward preferences. These details are essential for tailoring the training plan to the individual dog's needs while ensuring safety and effectiveness.

The *Outline Writer Agent* evaluates whether the behavior can be addressed using existing knowledge or if additional information is required. If necessary, it collaborates with the *Internet Research Agent* to gather relevant background knowledge or consults the *Behavior Requests Agent* to obtain further clarification from the handler. This process ensures that the draft outline is both well-informed and contextually relevant. After drafting the outline, the *Dog Details Agent* collects specific information about the individual dog that could influence the training plan. For instance, when creating a plan to extend the duration of a “sit” behavior, the agent may inquire about potential health concerns, such as hip issues, to ensure the plan is safe and achievable (Figure 8). Next, the *Evaluator* reviews whether the additional information gathered by the *Dog Details Agent* necessitates modifications to the draft outline. If revisions are needed, the *Outline Writer* updates the plan, and this cycle repeats until the draft accurately accounts for the dog's unique needs and circumstances.

Once the outline is finalized, the *Plan Writing Team* begins its work. The *Team Manager Agent* extracts individual training steps from the outline and assigns them to the appropriate *Specialist-Welfare Teams*, such as those focused on distractions, cues, duration, or distance. Each team generates a detailed training plan for its assigned step, incorporating welfare standards and individual dog characteristics. The completed plans are collaboratively stored in shared memory, ensuring accessibility for further refinement or review. The *Final Plan Writer* creates an overarching overview of the training steps, providing clear references to each detailed plan generated by the Specialist-Welfare Teams. Due to the inherent token length limitations of LLM responses, even in models with higher limits, the detailed plans for individual training steps are not merged into a single document. Instead, the overview links to the individual plans, enabling trainers to navigate the information efficiently while maintaining modularity. Although this design may result in some duplication across plans, it prioritizes clarity and accessibility. Future advancements, such as fine-tuning LLMs to handle longer outputs, could address this limitation (22). The *Plan Filer Agent* consolidates the finalized plans into the system's storage, ensuring they are stored and organized for immediate use. This step guarantees that trainers can easily access the complete set of plans when needed. Illustrative examples of the generated plans are provided in the Supplementary material Sections 1.2 and 1.3. They demonstrate the level of specificity the system can achieve. Each plan outlines precise training steps and includes actionable instructions tailored to individual training goals and dog characteristics. These examples also reflect the system's ability to generate collections of progressive steps, not merely isolated exercises. As described in the introduction, such structured progressions are fundamental to effective animal training.

In summary, this integrated workflow demonstrates the potential of agentic systems to produce detailed, actionable training plans. By leveraging modular agents and collaborative teams, the system ensures that the plans are progression-based and tailored to the specific needs of each dog. Furthermore, the workflow showcases the adaptability and scalability of agentic systems in addressing complex, real-world challenges, emphasizing their relevance in fields requiring precision and customization.

## 4 Discussion

This study demonstrates the feasibility of employing an agentic workflow to generate individualized and actionable training plans for dogs. It showed how modular agents can collaborate to address the complex task of creating detailed training steps while incorporating specific considerations, such as welfare compliance and health concerns. However, while this workflow offers a proof-of-concept, it is not intended as a one-size-fits-all solution. The diversity of tasks performed by dogs and the wide range of training methodologies used in the community make such a generalized solution impractical. Instead, this work highlights how the building blocks of an agentic workflow can be adapted to meet the specific needs of different tasks and training philosophies.

A major advantage of the presented approach lies in its modularity. By assigning distinct tasks to specialized agents, the workflow is both adaptable and efficient. Furthermore, each agent can operate independently using smaller LLMs, such as GPT-4o-mini^8^, which reduces operational costs compared to larger models. Importantly, this opens the door for workflows to utilize open-source LLMs, enabling deployment on local devices. Such an approach not only enhances data privacy but also allows users to operate the workflow without reliance on third-party providers, a significant consideration in sensitive applications. This modular design is further supported by a robust testing framework, which allows each agent and team to be tested individually and at different levels. These automated tests ensure consistent functionality even after modifications, such as replacing an LLM implementation or adapting an agent for a new task. By combining modularity with rigorous testing, the workflow maintains its reliability while remaining flexible and adaptable to future advancements in AI and machine learning.

One of the key strengths of the presented agentic workflow is its ability to generate actionable training plans with detailed progressions, outlining clearly defined steps for each stage of training. For instance, the *Duration Specialist Agent* does more than set the overarching goal of extending a behavior's duration–it provides a step-by-step progression plan, specifying each increment and the precise actions the handler should take to achieve success. This level of detail ensures that the plans are immediately usable by trainers, reducing ambiguity and eliminating the need for extensive interpretation. This capability addresses a notable limitation of general-purpose large language models (LLMs), which often produce generic, unstructured outputs that require significant adaptation to be practically useful. By leveraging modular agents designed for specific tasks, the workflow overcomes this gap, delivering training plans that are both precise and practical. This precision is especially critical in dog training, where clear, actionable steps are essential to achieving consistency and success.

Despite its strengths, the workflow also has limitations. One notable challenge arises from the tendency of LLMs to limit the length of their generated outputs, even when their theoretical token capacity is far greater. This affects the workflow when an agent is tasked with merging training plans from multiple specialist agents into a single, unified document. In such cases, the agent may inadvertently shorten or edit the detailed plans, potentially omitting important details or introducing inconsistencies. While fine-tuning an LLM to generate longer outputs could mitigate this issue (22), doing so would reduce the adaptability of the workflow by tying it to a specific model. As a result, the current implementation generates multiple separate files for the training plans, which, while ensuring completeness, can sometimes lead to overlapping or redundant training steps.

A critical aspect of transitioning the presented agentic workflow from a proof-of-concept to a robust, practically usable system involves addressing several usability and integration considerations. While the current implementation effectively demonstrates the feasibility and benefits of modular AI agents for generating detailed training plans, further advancements are necessary to facilitate broader adoption within the professional animal training community. Firstly, the current command-line-based interaction severely limits usability for typical dog trainers who may lack technical expertise. Thus, developing a user-friendly interface, preferably structured around a conversational or chat-like design, would significantly enhance accessibility. Such an interface would not only simplify interaction with the system but also serve as a repository to store and organize training plans, allowing trainers to revisit, adjust, and refine these plans over time. Secondly, enabling interactive dialogue with agents would allow trainers to iteratively revise and optimize training plans. Currently, interactions are primarily one-directional; however, incorporating interactive revision loops would enable trainers to clarify ambiguities, adjust recommendations, and ensure that the generated plans align closely with their practical needs and philosophies. Additionally, the usability of the system could be substantially enhanced by introducing persistent memory capabilities, such as those recently made available through LangGraph's LangMem module. This capability would allow the system to remember prior interactions and context-specific information about individual dogs, handlers, and training goals. Consequently, trainers would not need to repeatedly input foundational details, significantly reducing repetitive administrative tasks and streamlining the planning process. A further critical step toward practical adoption involves supporting gradual adaptation by allowing the incorporation and individualization of existing training plans. Rather than requiring trainers to start from scratch, the system could accept previously established plans and use its agents to tailor these plans to specific contexts, thus offering a gentler transition to the integration of AI-supported workflows. For deployment within organizations, it is essential to improve the integration of Standard Operating Procedures (SOPs). Utilizing advanced Retrieval-Augmented Generation (RAG) techniques would enable the system to dynamically extract relevant information from extensive SOP datasets and incorporate this information into training plan generation (20). Alternatively, the growing availability of large-context-window LLMs could facilitate direct incorporation of entire SOPs within queries, thus ensuring comprehensive adherence to organizational standards without the complexity of retrieval processes. Finally, integrating prior training data into the system presents an opportunity for significant advancement. Provided that suitable databases are available, agents could leverage historical training data to further customize and refine plans, enhancing predictive accuracy and responsiveness to individual dogs' evolving performance and behavioral patterns. Such integration would mark a substantial step toward realizing a truly adaptive and responsive training ecosystem. Collectively, these developments would transform the current agentic workflow from an innovative yet technical prototype into a comprehensive, intuitive, and adaptive system capable of providing meaningful, ongoing support to professional animal trainers.

Complementary to the technical enhancements described above, ensuring that the system meaningfully supports professional trainers in their day-to-day work also requires thoughtful integration into existing training practices. Rather than viewing deployment as a push for adoption, the focus should lie in identifying concrete, real-world contexts where the system can offer immediate and practical value. By embedding the technology into well-defined workflows–particularly in structured environments where training plans are already common–it becomes possible to align the agentic system with established routines and expectations. A promising entry point for such an approach lies within sub-communities that already rely on detailed, standardized training protocols. Examples include assistance dog programs or detection dog units, which often maintain well-documented progression plans and are accustomed to evaluating training outcomes systematically. These domains present an opportunity to pilot the system in collaboration with trainers who already work in a structured, goal-oriented manner and may therefore benefit from digital support tools without requiring major adjustments to their routines. In such a setting, one can begin by identifying a specific subset of the training process that involves frequent manual updates or adaptations–for example, adjusting exercises based on the dog's recovery status or environmental constraints. A tailored agent team can then be configured to support this particular task, leveraging the system's modular architecture. This targeted introduction allows for low-risk experimentation and provides trainers with an opportunity to explore how agentic workflows can enhance rather than replace their existing practices. Once familiarity and confidence have been established within this defined use case, the system could gradually be extended to cover additional aspects of the training workflow. This stepwise expansion supports both iterative refinement of the agents and the progressive adaptation of user workflows. It also fosters a sense of ownership among practitioners, as the system evolves in response to their needs and input rather than being imposed from the outside. Taken together, this gradual, context-aware strategy facilitates the integration of agentic workflows into the professional dog training landscape in a manner that is both respectful of established expertise and responsive to practical demands. By working in partnership with trainers, and adapting the technology to real-world use cases from the outset, the system may evolve into a trusted and flexible tool that complements existing methods–ultimately helping to reduce administrative workload and support more consistent, individualized training processes.

A broader and more fundamental challenge of the presented approach lies in evaluating the quality of the generated plans themselves. At present, a formal evaluation is not feasible due to the absence of a standardized “ground truth,” such as a database of universally accepted training plans. One potential evaluation approach could involve trainers reviewing and rating the generated plans; however, designing such a study as a double-blind experiment would be complex and resource-intensive. Additionally, the diversity of training philosophies within the community means that evaluation outcomes could be influenced by the individual perspectives of selected trainers. The most rigorous evaluation would involve training dogs using the AI-generated plans and comparing their outcomes to those trained with plans created by experienced humans. However, such an approach would require significant time and resources, which fall outside the scope of this manuscript. This gap highlights the need for broader discussions within the training community. While this manuscript does not propose a specific solution, fostering collaboration among trainers, researchers, and organizations could help establish benchmarks or shared evaluation criteria that respect the diversity of training philosophies and intellectual property. Thus, the presented workflow should be viewed as an initial prototype, demonstrating the potential of agentic workflows in this domain. Future studies should prioritize formal evaluations, comparing AI-generated plans with those of experienced trainers in controlled experiments. Such efforts would not only validate the quality of the plans but also provide valuable insights for further refinement and development.

Finally, the broader implications of agentic workflows warrant further exploration. Recent studies in reinforcement learning suggest that AI-designed training progressions can, in some cases, outperform those created by humans (23). This raises the intriguing possibility that AI-generated training plans could uncover novel and unconventional approaches to training behaviors–solutions that may not have been considered within traditional frameworks. Investigating whether these plans can introduce innovative techniques, while still aligning with established welfare standards and training philosophies, could open exciting new avenues for both research and practical application in animal training.

A fundamental aspect of this workflow is its role as a collaborative tool designed to assist, not replace, dog handlers. Just as AI-assisted tools in other fields, such as code generation or medical diagnostics, enhance human expertise rather than substitute for it, the outputs generated by this workflow are intended to support trainers. These plans should not be applied blindly; instead, they serve as a foundation that handlers can critically assess, adapt, and refine to suit their specific goals, training philosophies, and the unique needs of each dog. Beyond this, the workflow aims to free trainers from time-consuming administrative tasks, such as drafting detailed training plans from scratch. By accelerating the plan-writing process, the approach allows trainers to devote more time to what truly matters–interacting with and training their dogs. By placing the handler's expertise at the center of the process, the workflow reinforces the importance of professional judgment and experience. Its purpose is not to dictate how training should be conducted but to provide a flexible and innovative resource that empowers trainers to work more efficiently and effectively. In this way, the workflow complements the invaluable skills and insights of trainers, fostering a partnership between human expertise and AI-driven innovation.

To conclude, the ultimate vision for this work is the development of an integrated, AI-enhanced training ecosystem. In this envisioned system, training plans would be dynamically generated prior to each session, tailored to the dog's current behavioral status. During training, real-time data on the dog's progress and challenges could be used to iteratively update these plans, ensuring they remain responsive and effective. After each session, updated status information and identified challenges would be automatically incorporated into a centralized system, enabling continuous improvement over time. By combining data-driven refinements with real-time adjustments, this ecosystem has the potential to bridge the gap between cutting-edge technology and practical, humane training methodologies. While ambitious, this vision highlights the transformative possibilities of integrating AI with the art and science of dog training.

## Data Availability

The original contributions presented in the study are included in the article/Supplementary material, further inquiries can be directed to the corresponding author/s. The complete source code is available at https://github.com/Tier-Wohl-Team/AIAgents_TrainingPlan.

## Footnotes

^1^ https://openai.com/index/learning-to-reason-with-llms/

^2^ https://www.petprofessionalguild.com/barks/barks-magazine-blog/building-your-client-an-effective-pet-training-program

^3^ https://clickertraining.com/how-to-write-a-training-plan

^4^ https://www.karenoverall.com/wp-content/uploads/2020/06/Protocol-for-relaxation_Overall.pdf

^5^ https://www.guidedogs.com/puppy-raising-guidebook/beyond-puppy-raising/guide-dog-training/formal-guidework-training-phases

^6^ https://github.com/Tier-Wohl-Team/AIAgents_TrainingPlan

^7^ https://github.com/Tier-Wohl-Team/AIAgents_TrainingPlan/blob/master/README.md

^8^ https://openai.com/index/gpt-4o-mini-advancing-cost-efficient-intelligence/
