# Supplementary material for: On the potential of agentic workflows for animal training plan generation
Source: Front Vet Sci. 2025 May 20;12:1563233. doi: 10.3389/fvets.2025.1563233 (PMC12130733; doi:10.3389/fvets.2025.1563233)
Supplement: Supplementary file 1 [file Data_Sheet_1.pdf]

Supplementary Material

1 SUPPLEMENTARY DATA

1.1 Agents

1.1.1 Distraction Specialist Agent - Full Code

```
import textwrap

import dotenv
from langchain_core.messages import SystemMessage, HumanMessage
from langchain_openai import ChatOpenAI

from agents.BaseAgent import BaseAgent
from states.state_types import BehaviorState

dotenv.load_dotenv("TrainingPlan_Team/.env")


class DistractionSpecialist(BaseAgent):
    """Distraction Specialist
    This agent is responsible for training distractions.
    """
    NAME = "distraction_specialist"
    LLM_MODEL = "gpt-4o-mini"
    LLM = ChatOpenAI(temperature=0.0, model_name=LLM_MODEL)

    @staticmethod
    def action(state: BehaviorState):
        llm = DistractionSpecialist.LLM
        DistractionSpecialist.greetings()

        background_story = textwrap.dedent("""
            You are an experienced dog trainer having trained dogs to perform
   behaviours even under incredible
            distractions. In addition, you have a knack for helping novice trainers
   to teach their own dogs to cope
            with extreme distractions. The novice trainers love to work with you, as
    you give them extremely detailed
            instructions on how to start with mild distractions and build up from
   there in minuscule steps which enable
            the dog to master each training step successfully. You give the novice
   trainers a clear progression plan
            detailing out which distractions they should train, what the difficulty
   in this distraction is, when
            exactly they should reinforce the dog. In addition, each of your steps
   holds the information how the
```

```
        novice  trainer should react in case the distraction is too strong and
 the dog breaks the behaviour.
    """)
    task_prompt = textwrap.dedent("""
        Please write a training plan to assure that the behaviour {behavior} can
  be performed under distractions.
        The dog already knows the behaviour and can perform it. Only look at the
  distractions and leave other
        elements to the other members of your team.

        CURRENT STATUS

        {status}

        GOAL

        {goal}

        INFORMATION ABOUT THE DOG:

        {dog_details}

        In your training plan, start by stating the current status and then the
 goal. Next, develop a
        detailed progression plan for the trainer. In your
        progression plan, start from easy to hard. Clearly state the distraction
  and explain what the challenge is
        in this setup. When choosing distractions, take into account the
 information about the dog (if they are given)
        Also add the information how the trainer should react in case the
 distraction is too strong
        and the dog breaks the behaviour.

        If there is already a previous version of the plan, you can use it as a
 reference, but take into account
        the review from the welfare specialist if present.

        PREVIOUS VERSION

        {draft_plan}

        WELFARE REVIEW

        {welfare_review}


    """)

    messages = [
```

```
            SystemMessage(content=background_story),
            HumanMessage(content=task_prompt.format(
                behavior=state["behavior"],
                status=state["status"],
                goal=state["goal"],
                dog_details=state.get("dog_details", ""),
                draft_plan=state.get("draft_plan", ""),
                welfare_review=state.get("welfare_review", "")
            ))
        ]
        response = llm.invoke(messages)
        return {"draft_plan": response.content}
```

1.1.2 Distraction Specialist Agent - Example Output

Training Plan for "Sit" Under Distractions

CURRENT STATUS:
The dog stays sitting when the trainer moves their arms.

GOAL:
The dog stays sitting when a ball is thrown.

Progression Plan

Step 1: Mild Distraction - Trainer Clapping Hands

- **Distraction:** Trainer claps hands at a moderate volume.
- **Challenge:** The sound of clapping may catch the dog's attention, but it is a familiar and non-threatening noise.
- **Reinforcement:** If the dog remains in the sit position, reinforce immediately with a treat and praise.
- **If the dog breaks:** Calmly redirect the dog back to the sit position without scolding. Wait for a moment of calm before trying again.

Step 2: Moderate Distraction - Another Person Walking Nearby

- **Distraction:** A friend walks slowly past the dog at a distance of about 10 feet.
- **Challenge:** The movement of another person can be intriguing and may cause the dog to break the sit.
- **Reinforcement:** If the dog remains sitting, reinforce immediately. If the dog breaks, calmly guide them back to the sit position and try again.
- **If the dog breaks:** Use a gentle voice to redirect and encourage the sit. If the dog is too distracted, increase the distance between the dog and the person.

Step 3: Increased Distraction - Ball on the Ground

- **Distraction:** A ball is placed on the ground about 5 feet away from the dog.

- **Challenge:** The dog may want to chase the ball, which is a strong temptation.
- **Reinforcement:** If the dog stays in the sit position, reinforce immediately.
- **If the dog breaks:** Redirect the dog back to the sit position. If they are too focused on the ball, remove the ball and try again with a higher distance.

Step 4: Higher Distraction - Ball Thrown in the Air (No Movement)

- **Distraction:** The trainer throws a ball in the air but does not allow the dog to chase it.
- **Challenge:** The sight of the ball in motion can be very distracting.
- **Reinforcement:** If the dog remains sitting, reinforce immediately.
- **If the dog breaks:** Calmly guide the dog back to the sit position. If the dog is too distracted, reduce the distance of the throw or practice with a less exciting object.

Step 5: High Distraction - Ball Thrown with Movement

- **Distraction:** The trainer throws the ball and allows the dog to see it land.
- **Challenge:** The combination of movement and the ball landing can be very enticing for the dog.
- **Reinforcement:** If the dog remains sitting, reinforce immediately.
- **If the dog breaks:** Redirect the dog back to the sit position. If the dog is too distracted, practice with a less exciting object or throw the ball at a greater distance.

Step 6: Extreme Distraction - Multiple Balls Thrown

- **Distraction:** The trainer throws multiple balls in different directions.
- **Challenge:** The chaos of multiple balls can overwhelm the dog.
- **Reinforcement:** If the dog remains sitting, reinforce immediately.
- **If the dog breaks:** Calmly guide the dog back to the sit position. If the dog is too distracted, reduce the number of balls or practice with a single ball at a greater distance.

General Tips for the Trainer:

- Always start with the easiest distraction and gradually increase the difficulty.
- Use high-value treats for reinforcement, especially as distractions increase.
- Maintain a calm and positive demeanor, even if the dog breaks the behavior.
- Be patient and allow the dog to succeed at each level before moving on to the next.
- If the dog consistently breaks the sit, return to the previous step until they can perform reliably before progressing again.

By following this detailed progression plan, the trainer will help the dog learn to maintain the "sit" command even in the presence of significant distractions, ultimately achieving the goal of sitting while a ball is thrown.

1.2 Training Plan - Sit Duration

1.2.1 Overall Plan

Training Plan for Teaching Your Dog to Sit for 2 Minutes

Behaviour to Train: Sit
Current Status: The dog sits for 30 seconds
Goal: The dog sits for 2 minutes

Training Progressions:

- Gradually increase the duration from 30 seconds to 2 minutes in small increments.

Training Aids:

To successfully implement this training plan, you will need the following items:

1. **Treats:** Small, tasty treats that your dog loves. These will be used as positive reinforcement when your dog successfully sits for the desired duration.
2. **Clicker (optional):** A clicker can be used to mark the exact moment your dog successfully sits. This helps in reinforcing the behaviour.
3. **Leash:** A leash can help keep your dog in place during training sessions, especially if they are easily distracted.
4. **Quiet Training Area:** A space free from distractions (like other pets, loud noises, or people) to help your dog focus on the training.
5. **Water Bowl:** Ensure your dog has access to water, especially if you are training for an extended period.
6. **Comfortable Mat or Blanket (optional):** A designated spot for your dog to sit can help them understand where to stay during training.

Considerations:

- Ensure that the training environment is free from distractions to help the dog focus on the task.
- Use positive reinforcement, such as treats or praise, to encourage the dog during training sessions. This will help maintain motivation and make the training experience enjoyable.
- Monitor the dog's comfort level throughout the training. If the dog shows signs of stress or discomfort, take a break and reassess the training approach.

By following this plan and using the suggested training aids, you will be well on your way to helping your dog achieve the goal of sitting for 2 minutes!

1.2.2 Progressions Plan 1

Current Status: The dog has successfully learned to perform the 'sit' command for a duration of 30.0 seconds.

Final Goal: The objective is to extend the duration of the 'sit' command to 120.0 seconds.

Training Progressions:

1. **33.0 seconds:**
   - Start with 33 seconds.

- Repeat with 16 seconds.
- 5 seconds
- 24 seconds
- 45 seconds
- 1 second
- 16 seconds
- 8 seconds
- 33 seconds
- 45 seconds
- 3 seconds
- 16 seconds
- 10 seconds
- 33 seconds
- 5 seconds
- 24 seconds
- 45 seconds
- 16 seconds
- 1 second

2. **45.0 seconds:**

- Start with 45 seconds.
- Repeat with 22 seconds.
- 7 seconds
- 33 seconds
- 61 seconds
- 1 second
- 22 seconds
- 11 seconds
- 45 seconds
- 61 seconds
- 4 seconds
- 22 seconds
- 14 seconds
- 45 seconds
- 7 seconds
- 3 seconds

If the dog fails to perform a training step: If the dog is unable to maintain the 'sit' position for the designated duration, immediately return to the previous successful duration and practice that until the dog is comfortable. If the dog fails at more than three attempts at a specific duration, consider reducing the duration and gradually building back up to the target.

Considerations:

- Ensure that the training environment is free from distractions to help the dog focus on the task.
- Use positive reinforcement, such as treats or praise, to encourage the dog during training sessions. This will help maintain motivation and make the training experience enjoyable.

- Monitor the dog's comfort level throughout the training. If the dog shows signs of stress or discomfort, take a break and reassess the training approach.

1.3 Training Plan - Laser Directionals

1.3.1 Overall Plan

Final Training Plan: Teaching Your Dog to Go to a Target Using a Laser Pointer

Behavior to Train: Go to a target using a laser pointer
Current Status: No prior training with a laser pointer
Goal: The dog goes to a target at a distance of 50 meters

Training Outline

1. **Introduce the laser pointer to the dog, allowing them to explore and understand it as a tool.**
2. **Use the laser pointer to guide the dog to a target at a short distance (start with 5 meters).**
3. **Gradually increase the distance to the target in small increments, working up to 50 meters.**

Training Aids

To successfully implement this training plan, you will need the following materials:

- **Laser Pointer:** A safe, dog-friendly laser pointer that is easy to handle.
- **High-Value Treats:** Small, tasty treats that your dog loves to use as rewards during training.
- **Favorite Toys:** A toy that your dog enjoys playing with, which can also be used as a reward.
- **Clicker (optional):** A clicker can be used for marking desired behaviors if you choose to incorporate clicker training.
- **Training Leash:** A leash to help guide your dog during initial training sessions, if necessary.
- **Quiet Training Area:** A space free from distractions where you can focus on training.

Notes for the Novice Trainer

- Always monitor the dog's body language and adjust your approach based on their comfort level.
- Keep training sessions short and fun to maintain the dog's interest and enthusiasm.
- Be patient and consistent; every dog learns at their own pace.

By following this training plan, you will help your dog learn to associate the laser pointer with positive experiences and gradually increase their ability to follow it to a target at greater distances. For detailed instructions on each step, please refer to the accompanying detailed plans.

1.3.2 Progressions Plan 1

Training Plan for the Behavior: Explore the Laser Pointer

CURRENT STATUS:
The dog is familiar with the laser pointer.

GOAL:
The dog understands the laser pointer as a tool for play and exploration, rather than a source of frustration.

Progression Plan

Step 1: Introduce the Concept of Play with the Laser Pointer

- **Action:** Begin by using the laser pointer in a low-distraction environment. Shine the laser on the ground and encourage the dog to chase it. Use a happy, excited tone to engage the dog.
- **Reaction if Not Performing as Expected:** If the dog seems disinterested or confused, try moving the laser pointer more slowly or in a more predictable pattern. If the dog still does not engage, take a break and try again later.

Step 2: Pair the Laser Pointer with Positive Reinforcement

- **Action:** As the dog chases the laser, intermittently reward them with treats or praise when they successfully "catch" the light (e.g., when they touch the spot where the laser was). This helps the dog associate the laser pointer with positive experiences.
- **Reaction if Not Performing as Expected:** If the dog does not seem to understand the reward system, pause the session and try using a different toy or treat to regain their interest. Reintroduce the laser pointer after a short break.

Step 3: Increase Engagement with Interactive Play

- **Action:** Start moving the laser pointer in a way that encourages the dog to explore different areas (e.g., around furniture, up and down stairs). This will help the dog learn to engage with their environment while playing.
- **Reaction if Not Performing as Expected:** If the dog appears frustrated or loses interest, reduce the complexity of the movements. Keep the laser pointer movements simple and gradually increase difficulty as the dog becomes more engaged.

Step 4: Introduce a "Catch" Command

- **Action:** As the dog becomes more comfortable with the laser pointer, introduce a command like "Catch!" when you shine the laser. This will help the dog understand that they are expected to chase and engage with the light.
- **Reaction if Not Performing as Expected:** If the dog does not respond to the command, go back to rewarding them for chasing the light without the command. Gradually reintroduce the command once they are consistently engaging with the laser pointer.

Step 5: Establish Boundaries and Duration of Play

- **Action:** Set a time limit for laser pointer play (e.g., 5-10 minutes) to prevent frustration. End each session on a positive note, rewarding the dog with treats or a favorite toy after playtime.
- **Reaction if Not Performing as Expected:** If the dog shows signs of frustration or overstimulation, immediately stop the session and redirect their attention to a different activity or toy. Monitor their body language closely to ensure they are enjoying the play.

Step 6: Transition to Other Forms of Play

- **Action:** After the dog has learned to engage with the laser pointer positively, gradually transition to other forms of play (e.g., fetch, tug-of-war) to ensure they do not become overly fixated on the laser pointer.
- **Reaction if Not Performing as Expected:** If the dog seems reluctant to engage in other activities, continue to use the laser pointer as a reward for engaging in different play styles. This will help them understand that the laser pointer is just one of many fun tools.

Notes for the Novice Trainer:

- Always monitor the dog's body language and adjust your approach based on their comfort level.
- Keep training sessions short and fun to maintain the dog's interest and enthusiasm.
- Be patient and consistent; every dog learns at their own pace.

By following this progression plan, the novice trainer will help the dog understand the laser pointer as a tool for play and exploration, ensuring a positive and enriching experience.

1.3.3 Progressions Plan 2

Current Status: The dog has successfully learned to follow the laser pointer to a target at a distance of 5.0 meters.

Final Goal: The objective is to extend the distance of the 'follow the laser pointer to a target' behavior to 10.0 meters.

Training Progressions:

1. **5.0 meters:**
   - Start with 5 meters.
   - Repeat with 2 meters.
   - 6 meters
   - 3 meters
   - 7 meters
   - 3 meters
   - 5 meters
   - 1 meter
   - 7 meters
   - 4 meters
   - 3 meters
   - 5 meters
   - 1 meter

If the dog fails to perform at any distance, the trainer should repeat that specific distance until the dog successfully follows the laser pointer to the target. If the dog fails again at the same distance, the trainer should move on to the next distance in the progression. If the dog fails at more than 3 distances in a row, the trainer should revert to the previous progression to reinforce the behavior before attempting to increase the distance again.

Considerations:

- Ensure that the training environment is free from distractions to help the dog focus on the task.
- Use high-value treats or toys as rewards to motivate the dog during training sessions.
- Monitor the dog's body language for signs of stress or fatigue, and adjust the training duration accordingly to maintain a positive experience.
- If the dog shows signs of frustration or disinterest, take a break and return to a previously mastered distance before attempting to increase the distance again.

1.3.4 Progressions Plan 3

Current Status: The dog has successfully learned to follow the laser pointer to a target at a distance of 5.0 meters.

Final Goal: The objective is to extend the distance of the behavior 'follow the laser pointer to a target' to 50.0 meters.

Training Progressions:

1. **5.0 meters:**
   - Start with 5 meters.
   - Repeat with 2 meters.
   - 6 meters
   - 3 meters
   - 7 meters
   - 3 meters
   - 5 meters
   - 1 meter
   - 7 meters
   - 4 meters
   - 3 meters
   - 5 meters
   - 1 meter

2. **7.0 meters:**
   - Start with 7 meters.
   - Repeat with 3 meters.
   - 10 meters
   - 5 meters
   - 8 meters
   - 4 meters
   - 7 meters
   - 2 meters
   - 5 meters
   - 10 meters
   - 3 meters
   - 7 meters

- 2 meters

3. **10.0 meters:**

- Start with 10 meters.
- Repeat with 5 meters.
- 8 meters
- 4 meters
- 12 meters
- 6 meters
- 10 meters
- 3 meters
- 7 meters
- 10 meters
- 5 meters
- 12 meters
- 1 meter
- 10 meters
- 3 meters

4. **12.0 meters:**

- Start with 12 meters.
- Repeat with 6 meters.
- 10 meters
- 4 meters
- 15 meters
- 7 meters
- 12 meters
- 6 meters
- 15 meters
- 8 meters
- 12 meters
- 2 meters
- 10 meters
- 4 meters

5. **15.0 meters:**

- Start with 15 meters.
- Repeat with 7 meters.
- 12 meters
- 8 meters
- 13 meters
- 6 meters
- 20 meters
- 2 meters
- 10 meters
- 7 meters

- 15 meters
- 9 meters
- 20 meters
- 12 meters
- 8 meters
- 15 meters
- 7 meters
- 4 meters

6. **20.0 meters:**

- Start with 20 meters.
- Repeat with 10 meters.
- 12 meters
- 8 meters
- 15 meters
- 9 meters
- 17 meters
- 10 meters
- 25 meters
- 15 meters
- 3 meters
- 20 meters
- 10 meters
- 25 meters
- 13 meters
- 5 meters

7. **25.0 meters:**

- Start with 15 meters.
- Repeat with 25 meters.
- 12 meters
- 20 meters
- 5 meters
- 15 meters
- 25 meters
- 2 meters
- 18 meters
- 30 meters
- 12 meters
- 25 meters
- 15 meters
- 30 meters
- 25 meters
- 12 meters
- 3 meters

8.**30.0 meters:**

- Start with 25 meters.
- Repeat with 30 meters.
- 15 meters
- 20 meters
- 10 meters
- 17 meters
- 35 meters
- 5 meters
- 25 meters
- 30 meters
- 15 meters
- 20 meters
- 35 meters
- 17 meters
- 30 meters
- 15 meters
- 5 meters

9.**38.0 meters:**

- Start with 31 meters.
- Repeat with 38 meters.
- 19 meters
- 25 meters
- 12 meters
- 21 meters
- 44 meters
- 6 meters
- 31 meters
- 38 meters
- 19 meters
- 25 meters
- 44 meters
- 21 meters
- 38 meters
- 19 meters
- 6 meters

10.**48.0 meters:**

- Start with 39 meters.
- Repeat with 48 meters.
- 24 meters
- 31 meters
- 15 meters
- 26 meters

- 55 meters
- 8 meters
- 39 meters
- 48 meters
- 24 meters
- 31 meters
- 55 meters
- 26 meters
- 48 meters
- 24 meters
- 8 meters

If the dog fails to perform a training step: If the dog fails to follow the laser pointer to the target at any distance, repeat that specific distance until the dog successfully completes it. If the dog fails again, move on to the next distance in the progression. If the dog fails at more than three distances in a row, return to the previous progression and reinforce those distances before attempting to increase the distance again.

Considerations:

- Ensure that the training environment is free from distractions to help the dog focus on the task.
- Use high-value treats or toys as rewards to motivate the dog during training sessions.
- Monitor the dog's body language for signs of stress or fatigue, and adjust the training duration and intensity accordingly.
- Keep training sessions short and fun to maintain the dog's interest and enthusiasm.
